# 10-year experience in the use of Intravenous Immunoglobulin for Autoimmune Retinopathy

**DOI:** 10.1038/s41433-026-04368-6

**Published:** 2026-03-12

**Authors:** Alan R. Abraham, Lina Kobayter, Ester Carreno, Ian Yeung, Chrysanthi Tsika, Anthony G. Robson, Rebecca A. Baker, Carlos E. Pavesio, Andrew D. Dick, Colin J. Chu

**Affiliations:** 1 Bristol Eye Hospital, University Hospitals Bristol NHS Foundation Trust, Bristol, UK; 2 Academic Unit of Ophthalmology, University of Bristol, Bristol, UK; 3 Moorfields Eye Hospital NHS Foundation Trust, London, UK; 4 UCL Institute of Ophthalmology, London, UK; 5 NIHR Moorfields Biomedical Research Centre, London, UK

**Keywords:** Immunosuppression, Retinal diseases, Uveal diseases, Predictive markers, Prognosis

Autoimmune retinopathies which broadly overlap cancer associated retinopathy and non-paraneoplastic autoimmune retinopathy (npAIR) are complex diagnoses with visually devastating consequences [1]. There is no consensus on systemic immunosuppression, though a recent meta-analysis of case reports has suggested benefit [2]. We have seen limited response to classical immunosuppression in our practice and so have historically offered treatment with intravenous immunoglobulin (IVIg) which is commissioned in the UK, and here report on a cohort starting treatment over a 10-year period.

Testing for anti-retinal antibodies (ARAb) was performed in all 18 patients but even in those that were negative (5/18), if a clinical npAIR phenotype existed patients were still offered treatment with IVIg. Genetic testing was also undertaken (including whole exome sequencing in some patients) to exclude an inherited retinal disease explaining the phenotype. All had multidisciplinary evaluation including exclusion of an active underlying malignancy typically via oncology review and cross-sectional imaging (e.g., PET-CT and/or MRI). Our usual treatment approach was an empirical trial of systemic corticosteroids and mycophenolate mofetil. If a patient either failed to respond or was intolerant of these medications, we proceeded to IVIg therapy with a regime consisting of three infusions administered over 3 days, each of 0.5 g/kg [3, 4].

Each patient initiating IVIg was treated with 3 cycles of three infusions at 3–5-month intervals and their response to treatment was evaluated across multiple parameters including visual acuity, fields, retinal imaging and International Standard electroretinography (ERG) [5]. Response was defined pragmatically as the clinical consensus that visual and/or retinal function had stabilised sufficiently to justify continuation beyond three cycles; non-response was consensus of ongoing deterioration prompting cessation after three cycles.

Patients who continued IVIg therapy beyond the first 3 cycles were considered potential responders (Fig. 1). Of the cohort of 18 patients who initiated IVIg therapy, 10 continued beyond 3 cycles of IVIg. Of those continuing treatment, 4 of 9 patients had stabilised visual function at 1 year follow up but this diminished with time leaving only one patient on continued therapy. One patient showed further decline and referred for plasma exchange therapy but still experienced visual decline after this. Of the 9 patients who had more than three cycles, 5 showed continued decline in visual function during IVIg therapy. As shown in Table 1 ARAb was positive in 13/18 (72%), median IVIg duration was for 4 cycles (IQR 3–6) and only 1/18 (6%) continued IVIg with stable disease at time of last review.

**Fig. 1:**
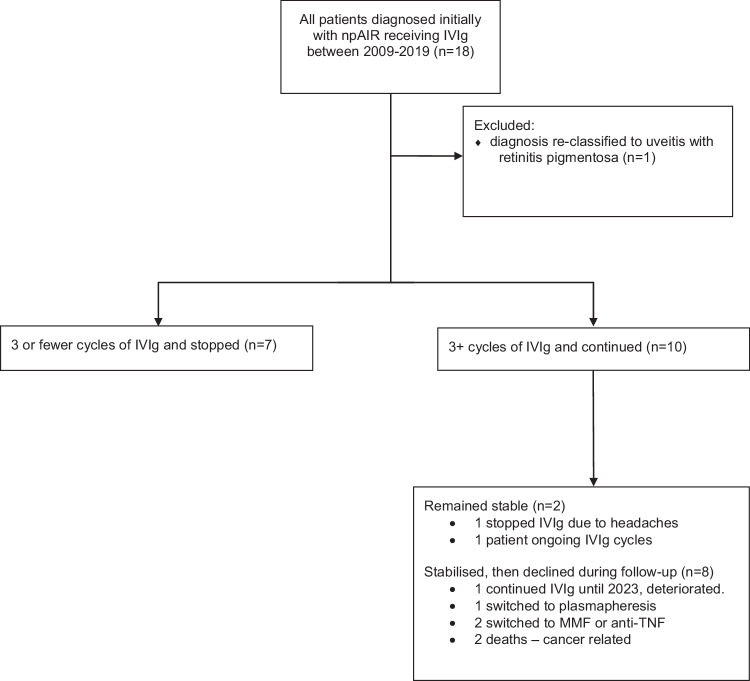
Outcome flowchart of patients initially diagnosed with non-paraneoplastic autoimmune retinopathy treated with intravenous immunoglobulin (IVIg).

**Table 1:** Table of key characteristics of patient cohort treated with IVIg for suspected autoimmune retinopathy.

Pt	ARAb status	ARAbs detected	Oncological history	Baseline cancer screening (PET-CT/MRI)	VA (baseline)	VA (last available)	PERG and ffERG post IVIg	Months of follow-up after end of IVIg cycles	IVIg cycles (n)	Clinical decision to continue treatment, last review
1	Positive	α‑enolase	None reported	PET-CT and MRI brain negative	RE 6/24; LE 6/18	RE 6/60; LE 6/60	Unrecordable	2	3	Discontinued after 3 cycles
2	Negative	Not detected	Right choroidal lymphoma treated with RTx (1995); NHL debulking + CHOP (1999); low‑grade follicular lymphoma	Baseline imaging not specified	RE HM; LE 6/24	RE NPL; LE HM	Unrecordable	N/A	2	Discontinued after 2 cycles Deceased (NHL)
3	Positive	40 kDa band	None reported	MRI brain negative; PET CT negative	RE 6/12; LE 6/5	RE 2/60; LE 6/6	RE unrecordable LE – partial, stable	N/A	2	Discontinued after 2 cycles
4	Positive	37 kDa (ROM1); α‑enolase; recoverin	None reported	PET CT negative	RE 6/24; LE 6/36	RE PL; LE HM	Unrecordable	11	3	Discontinued after 3 cycles
5	Positive	ROM1	Previous breast carcinoma, liposarcoma	PET-CT negative	RE 6/12; LE 6/12	RE 6/48; LE 6/48	pERG: partial, stable; ffERG: unrecordable	26	5	Discontinued after 5 cycles
6	Positive	28 kDa; 74 kDa bands	None reported	PET-CT negative	RE 6/4; LE 6/4	RE 6/9; LE 6/9	Unrecordable	12	3	Discontinued after 3 cycles
7	Positive	27, 30 (CA II), 36 (GAPDH), 40 (aldolase), 75 kDa bands	History of nasopharyngeal carcinoma	PET-CT: negative; MRI brain/orbits negative	RE 6/12; LE 6/9	RE 6/24; LE 6/60	Unrecordable	36	4	Discontinued after 4 cycles Deceased following aspiration pneumonia.
8	Negative	Not detected	None reported	PET-CT negative	RE 6/6; LE 6/9	RE 6/9; LE 6/9	Partial - stable	77	22	Continued (stable on IVIg)
9	Positive	23 (recoverin), 33 (enolase), 46, 60, 62 kDa bands	Not specified	Not specified	NR	NR	Unrecordable	14	4	Discontinued after 4 cycles
10	Positive	30; 72 kDa bands	Pituitary adenoma	MRI: no change in adenoma size; PET-CT negative	RE 2/60; LE 6/24	RE 1/60; LE 6/18	Unrecordable	37	5	Discontinued after 5 cycles, trialled plasma exchange – continued deterioration.
11	Positive	30; 33; 35; 45; 58 kDa bands	None reported	PET-CT negative; MRI brain/orbits negative	RE 6/12; LE 6/9	RE 6/18; LE 6/9	Partial - stable	46	15	Discontinued after 15 cycles, deteriorated.
12	Positive	Not specified	None reported	PET-CT negative	NR	NR	Partial - worse	33	6	Discontinued after 6 cycles (deterioration)
13	Positive	α‑enolase	None reported	Not specified	NR	NR	Partial - worse	N/A	2	Discontinued after 2 cycles (reclassified as retinitis pigmentosa)
14	Negative	Not detected	None reported	PET-CT negative	RE 6/36; LE 6/36	RE 6/36; LE 6/60	Partial - worse	N/A	2	Discontinued after 2 cycles (deterioration)
15	Negative	Not detected	None reported; incidental liver cyst under surveillance	PET-CT negative	RE 6/9; LE 6/5	RE 6/9; LE 6/6	Unrecordable	59	5	Discontinued after 5 cycles (deterioration)
16	Negative	Not detected	None reported	PET-CT: small chest nodule, stable vs. previous imaging; MRI brain negative	RE 6/12; LE 6/15	RE 6/24; LE 6/60	Partial - worse	13	3	Discontinued after 3 cycles
17	Positive	34; 44; 52; 58; 70; 136 kDa bands	Cervical carcinoma (chemo + brachytherapy + hysterectomy 5 y prior); breast carcinoma (mastectomy + tamoxifen 12 y prior)	PET-CT negative.	RE 6/6; LE 6/6	RE 6/6; LE 6/6	Partial - worse	38	14	Discontinued after 14 cycles (deterioration)
18	Positive	50 kDa band	Hodgkin lymphoma (LN excision + chemo 1990; relapse 2003 treated with ABVD chemotherapy)	PET CT negative	NR	NR	Partial - worse	23	6	Discontinued after 6 cycles Deceased from non-Hodgkin lymphoma.

*ARAb* anti‑retinal antibody, *ABVD* adriamycin/bleomycin/vinblastine/dacarbazine, *Aldolase* fructose‑bisphosphate aldolase, *CA II* carbonic anhydrase II, *CHOP* cyclophosphamide/doxorubicin/vincristine/prednisolone, *CT* computed tomography, *GAPDH* glyceraldehyde‑3‑phosphate dehydrogenase, *IVIg* intravenous immunoglobulin, *MRI* magnetic resonance imaging, *NAD* no abnormality detected, *NHL* non‑Hodgkin lymphoma, *PET-CT* positron emission tomography-computed tomography, *RTx* radiotherapy, *VA* visual acuity, *HM* hand movements, *PL* perception of light, *NPL* no perception of light, *NR* not recorded, *PERG* pattern electroretinogram, *ffERG* full field electroretinogram, *Y* years, *Unrecordable* no reproducible response, *Partial* recordable but reduced amplitude; stable/worse judged against laboratory repeatability limits or consensus where serial data incomplete.

In this study, treatment for npAIR was informed by multimodal monitoring including objective ERG evaluation of retinal function, but there is a clear need for further research into the underlying pathogenesis and to develop a clinically validated diagnostic test. In this cohort, ARAb status did not clearly identify sustained responders, supporting the possibility that ARAbs may not be the sole driver of disease in all patients. Until this cohort can be better defined clinicians will continue to face difficulty in giving patients a clear prognosis and selecting cases for immunomodulation such as IVIg, plasmapheresis, and biologic therapy [3].

## Data Availability

The datasets generated during and/or analysed during the current study are not publicly available due to the inclusion of confidential patient data. De-identified data may be made available from the corresponding author on reasonable request, subject to appropriate governance approvals.
